# Sulfur Mustard-induced Changes in Blood Urea Nitrogen, Uric Acid and Creatinine Levels of Civilian Victims, and Their Correlation with Spirometric Values

**Published:** 2018-11

**Authors:** Ensieh Sadat MIRSHARIF, Fatemeh HEIDARY, Mohammad Reza VAEZ MAHDAVI, Reza GHAREBAGHI, Shahriar POURFARZAM, Tooba GHAZANFARI

**Affiliations:** 1. Immunoregulation Research Center, Shahed University, Tehran, Iran; 2. International Virtual Ophthalmic Research Center, Tehran, Iran

**Keywords:** SM-exposed, Urea, Creatinine, Uric acid, Glomerular filtration rate

## Abstract

**Background:**

The aim of this study was assessment of the chronic effects of sulfur mustard (SM) among victims.

**Methods:**

In this cohort study, 355 SM-exposed subjects from Sardasht, and 123 controls from Rabat, both from West Azerbaijan Province, Iran were included. The spirometric evaluation and the global initiative for chronic obstructive lung disease (GOLD) classification were applied for all. Serum levels of urea, creatinine (Cr), and uric acid (UA) and glomerular filtration rate (GFR) were assessed. Data analysis was conducted using IBM SPSS.

**Results:**

All were male, with a mean age of 43.7±10.7 and 41.6±9.9 years in case and control groups, respectively. The case group had significantly higher values of Cr (*P*<0.001) and UA (*P*=0.018) than the control group. This was also the case in the Cr level (*P*<0.001) in subjects without pulmonary dysfunction, between both groups. There was significant difference in the GFR (*P*=0.047) between both groups and between subgroups with pulmonary dysfunction in the case and control groups (*P*=0.045), as well as between SM-exposed subjects with and without pulmonary dysfunction (*P*=0.009). Serum Cr, UA, sUA/Cr ratio, and BUN as well as the GFR did not have any significant correlation with forced expiratory volume in one second (FEV1), forced vital capacity (FVC), and FEV1/FVC ratio.

**Conclusion:**

Despite significantly high levels of Cr and UA in the case group, no significant correlation was found between serum Cr, UA, sUA/Cr ratio, BUN, and GFR with spirometric values. Further studies are required to reveal the underlying molecular and clinical significance of these findings.

## Introduction

Exposure to Sulfur mustard (SM) as a chemical blistering weapon could induce a variety of manifestations in mankind including dermal, ocular, pulmonary, gastrointestinal, hematological, and reproductive toxicity (1, 2). The molecular pathogenesis is the impairment of the major cellular components such as DNA, RNA, lipids, carbohydrates, and proteins, leading to biological consequences in the majority of tissues in the body (3, 4). Graphical studies of the whole body have detected SM in the liver and kidneys, following intravenous and percutaneous injections (5).

The kidney could be affected by reactive oxygen species (ROS) due to the profound amount of long-chain polyunsaturated fatty acids (LCPs) in renal lipids. Therefore, alkylating of subcellular components following SM exposure leads to metabolic disruption and induces oxidative stress in the kidney. A molecular chain of this induced oxidative stress causes the formation of ROS, lipid peroxidation, and protein oxidation, and sets up a rise in antioxidant enzymes, such as glutathione-S-transferase, superoxide dismutase, and catalase (6).

Serum concentrations of creatinine (Cr), uric acid (UA), and Blood urea nitrogen (BUN) reveal renal function. Among them, Cr is the one most commonly used for evaluation of renal dysfunction (7). Moreover, UA is used not only for the assessment of renal function but also considered to be a marker reflecting hypoxic status (8). Alongside with application of these markers for renal function, currently growing numbers of studies evaluating serum UA to Cr ratio (sUA/Cr ratio) as a marker in pulmonary disorders such as Chronic Obstructive Pulmonary Disease (COPD). A higher level of this ratio was associated with abnormally low spirometric values and a high level of dyspnea. In addition, this ratio was higher in COPD patients than in healthy subjects (9, 10).

Despite extensive research for evaluating the dermal, pulmonary, and ocular complications of SM toxicity, there are few studies about its renal side effects. To the best of our knowledge, no study has been conducted yet to assess the correlation between renal function biomarkers with spirometric values in SM-exposed patients.

Therefore, the aim of current study was to assess the chronic effects of SM in victims of this warfare agent, in the 20 years after the first exposure.

## Materials and Methods

### Study Design, Participants, and Clinical Evaluation

We have explained study design in detail in our publication on the Sardasht-Iran Cohort Study (SICS) (11). We included 355 SM-exposed subjects from the city of Sardasht (Sardasht County, West Azerbaijan Province, Iran) and 123 control subjects from the city of Rabat (Central District of Sardasht County, West Azerbaijan Province, Iran) in 2006, after a full explanation to all subjects and signing of written consent by all.

An approval of this study was received from the Ministry of Health and Medical Education (Iran) and the Research Board for Ethics in Research Center for Janbazan Medical and Engineering.

All subjects underwent spirometric evaluation based on the American Thoracic Society criteria (12), using spirometry (HI-801; Central Sports, Tokyo, Japan) by a senior trained nurse. The measurements were repeated three times for each case and the best one was chosen for the final analysis. Global initiative for chronic obstructive lung disease (GOLD classification) was applied to categorize the severity of pulmonary function in the subjects (13, 14).

### Serum Collection and Measurement of Biomarkers

With use of a 21-gauge needle blood sample was taken and placed into Vacutainer blood collecting tubes (BD Biosciences). Following 20 min of centrifugation of the blood samples at 2000×g (4 °C), serum was retrieved, aliquoted, and frozen at −80 °C for future laboratory measurements of biomarkers. Serum levels of urea, Cr, and UA were assessed by suitable assay kits (Pars Azmoon-Co, Tehran, Iran). Values of urea were converted to BUN for better clinical evaluation.

### Statistical Analysis

BUN, Cr, UA, and the sUA/Cr ratio were considered to be the biochemical parameters. The glomerular filtration rate (GFR) was calculated for each study group using the Cockcroft-Gault (CG) formula(15). Mean and standard deviation (SD) were calculated. T-test used for statistical comparison between two groups. The correlation between biochemical factors and pulmonary dysfunction was computed with the Pearson correlation coefficient. A Receptor Operation Curve (ROC) analysis was done to evaluate the performance of biochemical factors in the detection of pulmonary dysfunction. The Area under the curve (AUC) and the significance of this analysis were reported. Sensitivity and specificity were reported for biochemical parameters. Significance level defined as P-values less than 0.05. Data analysis was performed using IBM SPSS 21 (Chicago, IL, USA).

## Results

### Comparison of serum levels of BUN, Cr, UA, and GFR among two groups with and without pulmonary dysfunction

Out of 478 study subjects, 355 were from the case group (the SM-exposed) and 123 were from the control group. All subjects were male, with a mean age of 43.7±10.7 years in the case group and 41.6±9.9 years in the control group. The case group had significantly higher values of Cr (*P*<0.001), UA (*P*=0.018), and BUN (*P*=0.018) as compared to the control group. This significant difference was noted in the Cr level (*P*<0.001) in subjects without pulmonary dysfunction, between two groups. Subjects in the case group had an insignificant lower serum sUA/Cr ratio (*P*=0.393) (Table 1).

**Table 1:** Comparison of the serum levels of BUN, Cr, UA and GFR in study groups with and without pulmonary dysfunction; Using GOLD stage for disease severity

	***Pulmonary result GOLD***	***Control***			***Median***	***SM-exposed***			***Median***	***P-value***
		***N***	***Mean***	***SD***		***N***	***Mean***	***SD***		
Cr mg%	Without pulmonary dysfunction	110	1.00	0.15	1.00	297	1.08	0.18	1.10	<0.001^*^
	With pulmonary dysfunction	13	1.03	0.11	1.00	58	1.12	0.16	1.10	0.080
	Total	123	1.00	0.14	1.00	355	1.08	0.17	1.10	<0.001^*^
	*P*-value		0.476				0.139			
UA mg%	Without pulmonary dysfunction	110	5.70	1.10	5.7	297	5.90	1.20	5.7	0.060
	With pulmonary dysfunction	13	5.40	0.90	5.6	58	6.00	1.10	6.0	0.085
	Total	123	5.60	1.00	5.7	355	5.90	1.20	5.8	0.018^*^
	*P* -value		0.399				0.650			
sUA/Cr ratio	Without pulmonary dysfunction	110	5.76	1.39	5.55	297	5.60	1.49	5.33	0.340
	With pulmonary dysfunction	13	5.25	0.90	5.27	58	5.45	1.24	5.53	0.591
	Total	123	5.70	1.35	5.50	355	5.58	1.45	5.36	0.393
	*P* -value		0.202				0.458			
BUN	Without pulmonary dysfunction	110	13.98	5.41	13.08	297	13.86	3.51	13.55	0.060
	With pulmonary dysfunction	13	12.29	2.86	12.15	58	13.17	2.97	12.62	0.085
	Total	123	13.80	5.22	13.08	355	13.74	3.43	13.08	0.018
	*P* -value		0.399				0.650			
GFR	Without pulmonary dysfunction	111	103.2	26.6	100.8	292	99.4	27.1	96.1	0.205
	With pulmonary dysfunction	13	104.0	28.9	102.9	58	89.3	23.6	89.0	0.045^*^
	Total	124	103.3	26.7	100.9	350	97.7	26.7	94.6	0.047^*^
	*P* -value		0.918				0.009^*^			

GOLD= Global Initiative for Chronic Obstructive Lung Disease; Cr= Creatinine; UA= Uric acid; sUA/Cr ratio= Serum Uric acid to creatinine ratio; BUN= blood urea nitrogen; GFR: glomerular filtration rate; N= number; SD= standard deviation; mg: milligram.

* *P* value less than 0.05

Using an independent t-test, a significant difference was revealed in the GFR (*P*=0.047) between the study groups (Table 1).

This was also the case between subgroups with pulmonary dysfunction in the control and case groups (*P*=0.045), and between case group with and without pulmonary dysfunction (*P*=0.009).

### Correlation of serum levels of BUN, Cr, UA, and GFR with spirometry values

Serum Cr, UA, sUA/Cr ratio, BUN, and GFR did not have any statistically significant correlation with all measured spirometric values (Table 2). These values included FEV1 (Forced Expiratory Volume in One Second), FVC (Forced Vital Capacity), and FEV1/FVC in both groups.

**Table 2:** Correlation between serum Cr, UA, sUA / Cr ratio, BUN and GFR with spirometry values

***Variable***		***Control***			***SM-exposed***		
		***FVC %***	***FEV1 %***	***FEV1/FVC %***	***FVC %***	***FEV1 %***	***FEV1/FVC %***
Cr mg%	r	-	0.023	0.014	0.021	0.000	−0.076
		0.004					
	*P*-value	0.964	0.804	0.913	0.697	0.993	0.261
UA mg%	r	0.098	0.066	0.189	−0.054	−0.091	−0.049
	*P*-value	0.281	0.469	0.125	0.306	0.088	0.464
sUA/Cr ratio	r	0.080	0.042	0.133	−0.051	−0.058	0.020
	*P*-value	0.379	0.648	0.283	0.335	0.277	0.765
BUN	r	0.098	0.066	0.189	−0.054	−0.091	−0.049
	*P*-value	0.281	0.469	0.125	0.306	0.088	0.464
GFR	r	0.015	−0.082	−0.067	0.045	0.036	0.023
	*P*-value	0.870	0.368	0.591	0.399	0.498	0.735

Abbreviations: Cr= Creatinine; UA= Uric acid; sUA/Cr ratio= Serum Uric acid to creatinine ratio; BUN= blood urea nitrogen; GFR: glomerular filtration rate; FVC= Forced vital capacity; FEV1= forced expiratory volume in one second

### Performance of biochemical factors; sensitivity and specificity in detection of pulmonary dysfunction

**Fig. 1:**
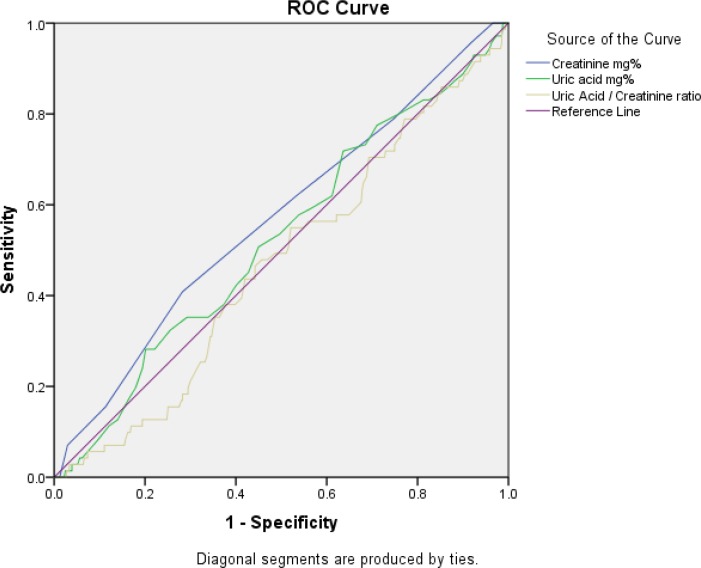
Receptor Operation Curve (ROC) curve for evaluating performance of biochemical factors in the detection of pulmonary dysfunction

A ROC analysis was done in evaluating the performance of biochemical factors in the detection of pulmonary dysfunction (Fig. 1). The AUC and significance of this analysis (*P*-value) were 0.567 (*P*=0.07), 0.524 (*P*=0.527), and 0.469 (*P*=0.409) for Cr, UA and the sUA/Cr ratio respectively. The largest AUC belonged to Cr and none of them were significantly related to the presence of pulmonary dysfunction.

For pulmonary dysfunction based on GOLD classification, UA alone was more specific than the sUA/Cr ratio, with similar sensitivity at higher cutoff values and more sensitivity with similar specificity at lower cutoff values (Table 3, 4). This was applicable to all study groups.

**Table 3:** Cutoff values of Uric Acid ratio for pulmonary dysfunction (GOLD)

***Positive if ≥ Values***	***Sensitivity***	***Specificity***
	Uric Acid^*^	
3.00	1	0.002
3.55	0.972	0.022
4.05	0.93	0.052
4.55	0.873	0.118
5.05	0.803	0.236
**5.35**	**0.718**	**0.364**
5.55	0.592	0.435
6.05	0.423	0.6
6.55	0.282	0.779
7.05	0.127	0.86
7.55	0.042	0.939
8.05	0.028	0.961
8.60	0	0.978
9.05	0	0.988

Note: ^*^The Area under the curve (AUC): 0.524, 95% Confidence interval (CI): 0.451–0.596

**Table 4:** Cutoff values of Serum Uric acid to creatinine ratio for pulmonary dysfunction (GOLD)

***Positive if ≥ Values***	***Sensitivity***	***Specificity***
	Serum Uric acid to creatinine ratio^*^	
3.13	1	0.005
3.54	0.944	0.022
4.04	0.887	0.091
4.45	0.803	0.187
5.04	0.577	0.351
5.50	0.493	0.514
**5.57**	**0.465**	**0.558**
6.0	0.254	0.678
6.52	0.127	0.806
7.0	0.07	0.862
7.56	0.056	0.926
8.06	0.028	0.936
8.52	0.028	0.956
9.14	0.014	0.968
9.56	0	0.975

Note: ^*^The Area under the curve (AUC): 0.469, 95% Confidence interval (CI): 0.399–0.5398

## Discussion

In this study, the difference in the sUA/Cr ratio between two groups was insignificant. However, the levels of Cr, UA, and BUN were significantly higher in the case group. This significant difference seen in the Cr levels of subjects without pulmonary dysfunction, between two groups.

In addition, there were significant differences in the GFR between study groups among subgroups with pulmonary dysfunction in control and SM-exposed groups, and also between the case group with and without pulmonary dysfunction.

Subjects in the case group had insignificantly lower sUA/Cr ratios. The AUC and the significance of this analysis showed that there is an insignificant performance of Cr, UA, and UA/Cr in the presence of pulmonary dysfunction. Serum Cr, UA, sUA/Cr ratio, and BUN did not have any significant correlation with FVC, FEV1, and FEV1/FVC in both groups.

Studies have shown both short- and long-term side effects in a variety of body organs after SM exposure (16). Similarly, our results showed significantly higher levels in renal function biomarkers, including Cr, UA, and BUN, as well as reduced GFR in the case group, as compared to the control group. Abundance of LCPs in the renal tissue makes this vital organ susceptible to ROS. Therefore, SM-induced oxidative stress led to an increase in the Cr level and a reduction of GFR, which by itself could impair renal function (6). GFR reduction in chronic kidney disease (CKD) frequently causes Hyperuricemia (HUA). Since two-thirds of daily UA excretion is through the renal tissue, more than 90% of HUA is caused by renal impairment. Studies revealed that induced oxidative stress following the rise in UA level may lead to glomerular and systemic hypertension (17).

Despite a comprehensive number of reported studies about the short-term effects of SM on various organs (18–23), few studies have been conducted on the long-term effects of exposure to this gas on organs, mainly the kidneys. The delayed biochemical complications of SM were investigated in 42 severely toxic veterans whose disability was more than 40%, 23 years after exposure. BUN, Cr, and UA levels did not show any significant differences between the case and control groups. However, significant reductions in serum albumin and total protein levels were found between the two groups. This reduction could be due to renal losses (16). In contrast, in current study, there were significantly higher levels of Cr, UA, and BUN in the case group than the control group. This contradiction may lie in the recruitment of their study subjects—who were severely injured—in contrast to our subjects. Furthermore, we found significant differences in the GFR between the study groups, parallel to higher values of serum biomarkers, which could justify the significantly higher levels of Cr, UA, and BUN in our study. However, the former study did not present the GFR of their study subjects, which makes it impractical to have further comparisons and explanation of differences between the findings of the two studies.

The renin-angiotensin-aldosterone system (RAAS), having various regulatory roles in the body, is closely related to renal and pulmonary functions (24). Serum uric aicd and sUA/Cr ratio were significantly higher in patients with COPD with systemic disease like hypertension and malignancy, compared to healthy controls (10). Moreover, their ROC curve analysis revealed the importance of the sUA/Cr ratio in prediction of COPD exacerbation, especially in higher values, as compared to the sUA level per se. In contrast, despite significantly higher levels of UA and Cr in the case group than the control group, we did not find a significant link between the sUA/Cr ratio with pulmonary dysfunction and spirometric values in both groups. Although the ROC curve analysis showed high specificity in the higher cutoff value and higher sensitivity in the lower cutoff value for the UA level as compared to the sUA/Cr ratio, the UA level had no diagnostic value because of the insignificance of these results.

The chronic obstructive pulmonary disease patients, with a sUA/Cr ratio higher than median value, had lower FVC and FEV1 spirometric values and a higher rate of dyspnea (9). The sUA/Cr ratio correlated with FVC, FEV1, and dyspnea, and also, that this ratio could be considered a valuable predictive parameter in COPD patients. In our study, despite a significantly higher level of sUA in the case group than the control group, no significant relationship has been noted with the sUA/Cr ratio in both groups. Moreover, we did not find any significant relation between spirometric values and serum levels of UA, Cr, and the sUA/Cr ratio. This difference could be because of the distinct underlying pathophysiology mechanisms of SM-induced pulmonary dysfunction and COPD.

The effect of acute nitrogen mustard exposure (NM, an analogue of SM) on the kidneys of mice was evaluated and a significant decrease in kidney weight and serum Cr level together with a significant rise in BUN and BUN to Cr ratio was found. Tissue catabolism or starvation may lead to Cr reduction and that pre-renal azotemia caused a significant rise in BUN and BUN to Cr ratio (25). In current study, we found significantly higher levels of Cr, UA, BUN, and GFR, in conjunction with an insignificant lower sUA/Cr ratio, in the case group. In fact, different pathophysiological manifestations of acute NM-exposure (in the former study) and chronic SM-exposure (in the latter study) could explain the differences in blood biomarkers in these two studies. Moreover, these contrary levels were seen in inflammatory biomarkers following acute (26,27) and chronic (28–30) SM exposure.

Renal structure could be injured by inflammatory mediators or immune-mediated factors linked with primary lung pathology. Otherwise, quite the opposite, it could be kidney disease that determines successive pulmonary damage. Hight rate of chronic renal failure (CRF) and microalbuminuria among COPD patients has been reported, lately. Which may suggest a correlation between these two entities. Moreover, levels of Cr, cysteine, and brain natriuretic peptide were significantly high in COPD subjects (31).

Studies revealed a higher risk of CRF in COPD patients and acute kidney injury (AKI) following lung transplantation and a strong positive correlation was noted between the serum Cr level and pulmonary function (32–35). In contrast, in current study, in spite of significantly higher levels of UA, Cr, and BUN in the case group, no significant relation of serum Cr, UA, sUA /Cr ratio, BUN, and GFR with pulmonary dysfunction and all measured spirometric values was found in both groups.

By evaluating the gender difference in correlation between pulmonary function abnormalities and eGFR using spirometric parameters, a parallel reduction was found in eGFR with a reduction in FEV1/FVC in both sexes and a reduction in FVC in males (36). Although, we found a significant difference in the GFR between the two groups and between subgroups with pulmonary dysfunction in both groups, the correlation between the GFR and spirometric parameters was insignificant.

Other studies have shown a reduction in the GFR following an increase in UA and Cr levels, which is parallel with the findings of our study (17, 37). An increase in UA level by inducing oxidative stress and endothelial dysfunction led to systemic and glomerular hypertension, which subsequently causes reduced renal blood perfusion and GFR which, decreases renal function (17). This postulated pathophysiological mechanism may account for changes in renal function biomarkers and reduction in the GFR of our SM-exposed subjects.

## Conclusion

Despite significantly higher levels of Cr and UA, and a significant reduction in the GFR of the case group than the control group, no significant correlation was found between serum Cr, UA, sUA/Cr ratio, BUN, and GFR with spirometric values. Further studies are required to reveal the underlying molecular and clinical significance of these findings.

## Ethical considerations

Ethical issues (Including plagiarism, informed consent, misconduct, data fabrication and/or falsification, double publication and/or submission, redundancy, etc.) have been completely observed by the authors.
